# The knowledge-risk-behaviour continuum among young Ugandans: what it tells us about SRH/HIV integration

**DOI:** 10.1186/s12889-019-6809-y

**Published:** 2019-05-29

**Authors:** Raquel Palomino González, Damazo Twebaze Kadengye, Roy William Mayega

**Affiliations:** 1 United Nations Population Fund (UNFPA), Kampala, Uganda; 2 African Population and Health Research Center, Manga Cl, Nairobi, Kenya; 3 School of Public Health, Makerere University, Kampala, Uganda; 4 School of Statistics and Planning, Makerere University, Kampala, Uganda

**Keywords:** SRH, HIV, Knowledge, Risk perception, Sexual behaviour, Young people, Uganda

## Abstract

**Background:**

With a human immunodeficiency virus (HIV) prevalence of 2.1% among 15–24 year olds, opportunities for further integration of sexual and reproductive health (SRH) and HIV prevention services for young people in Uganda exist. We examine a range of factors that contribute to variance in risky sexual behaviour among young Ugandans.

**Methods:**

A cross-sectional, nationally representative household survey was conducted between February and March 2016. The questionnaire used assessed knowledge, attitudes and practices related to SRH among young people aged 10–24 years. A composite indicator was constructed to assess risky sexual behaviour, defined as being involved in sexual relations under the influence of alcohol or drugs, engaging in sexual activities without a condom, and having multiple sexual partners in the 6 months preceding the survey. Exploratory analysis was conducted to provide descriptive statistics. Logistic regression was conducted to determine the factors associated with risky sexual behaviour. This analysis focuses on the sub-category aged 15–24 years, comprised of 2725 respondents.

**Results:**

Knowledge levels of family planning (FP), sexually transmitted infections (STIs) and HIV across all respondents were high (above 82%). Self-reported perceived risk of STIs and pregnancy was consistently higher among 20–24 year old respondents, with 61.5% feeling at risk of STIs compared to 46.2% of 15–19 year olds. A total of 22.7% of respondents reported having been involved in risky sexual behaviour. Factors associated with risky sexual behaviour among the 15–19 years group included gender, single orphanhood, casual work, schooling status, FP knowledge and self-perceived risk of STIs/HIV. For the 20–24 year old respondents, significantly associated factors included gender, educational level, relationship to head of household, place of residence, and self-perceived risk of pregnancy.

**Conclusions:**

Despite high general SRH/HIV knowledge and perceived risk of pregnancy and HIV, risky sexual behaviour among young Ugandans remains high. Effectiveness gaps in the integrated SRH/HIV response for young people should be addressed and targeted interventions focused on holistic prevention at individual level through information, risk awareness, and skill development should be combined with interventions targeting social structures affecting individual behaviour.

**Electronic supplementary material:**

The online version of this article (10.1186/s12889-019-6809-y) contains supplementary material, which is available to authorized users.

## Background

The possible benefits derived from linkages between SRH and HIV at the policy, systems and service delivery levels are today widely recognized [1–6]. The renewed emphasis on the SRH/HIV integration agenda, which seeks to reshape health service provision for SRH and HIV services by providing comprehensive health services and referrals in this area [7], can be an effective way of accelerating the achievement of 90–90-90 targets [8] and advancing the Sustainable Development Goal (SDG) 3 target to end the AIDS epidemic by 2030 [9].

For young people in particular, SRH/HIV integration is expected to deliver increased uptake of both SRH and HIV services, improved knowledge of HIV status, promotion of safer sex, reduction in HIV-related stigma and discrimination, better client experience and improved satisfaction, greater support for dual protection and better understanding and protection of individuals’ rights [1, 10]. In Uganda, SRH/HIV integration has taken place at policy, systems and service delivery level since 2012, when the AIDS Control Programme and the Reproductive Health Division of the Ugandan Ministry of Health were mandated to advance this agenda [11]. Integration of SRH and HIV into all care and treatment services was reflected as a strategic objective of the National Strategic Plan for HIV&AIDS 2011/12–2014/15 [12], and from then onwards in the major policies, strategies and guidelines dealing with SRH and HIV in the country [13, 14].

The effectiveness of the SRH/HIV integrated response achieved at national level can be measured using the list of indicators developed by the Interagency Working Group on SRH and HIV Linkages across 8 different domains. Indicators include, among others, the percentage of adults aged 15–49 who have more than 1 sexual partner in the past 12 months and report use of condom during last sex, and the HIV prevalence rate among young people [15]. When looking at how Uganda performs on these indicators, and despite the government commitment to accelerate the rollout of this agenda at both national and local levels, significant gaps still exist in relation to SRH/HIV outcomes among young people.

An estimated 67 new young Ugandans get infected with HIV every day. This represents 44.1% of all new infections in the country, the majority of which are sexually transmitted [14]. The HIV prevalence rate among those aged 15–24 years stands at 2.1% [16]. Young women in particular are at significant risk for both unintended pregnancy and HIV infection. HIV prevalence among 15–24 year olds is 4 times higher among women (3.3%) than men (0.8%) [16], and 23.5% of young girls aged 15–19 years have given birth [17].

Significant grounds for further integration of SRH/HIV services for young people in Uganda still exist [18]. Empowering young Ugandans to reduce HIV risk exposure is a fundamental deliverable for SRH and HIV integration, and a more detailed understanding of key factors that contribute to variance in SRH/HIV risk behaviour among young Ugandans can help design interventions that effectively reduce HIV risk exposure among this target group. The objective of this study was therefore to establish knowledge levels, attitudes and practices related to sexual and reproductive health among young people in Uganda.

## Methods

### Study design

A cross-sectional household survey was conducted in Uganda between February and March 2016. The study targeted a nationally-representative sample of households calculated based on the UN Department of Economic and Social Affairs’ practical guidelines on designing household survey samples [19]. The overall household sample size was 2976 households. Sample size calculations were based on the following assumptions: confidence level set at 95%, percentage of young people age 10–24 who have comprehensive knowledge about HIV/AIDS set at 40%, design effect of 2.0, non-response rate of 20%, a margin of error/precision of 10% and an estimated average 1.48 respondents aged 10–24 years per household.

Given the research questions selected for this paper which focus on sexual activity and SRH/HIV knowledge, the study sub-category aged 15–24 years comprised of 2725 respondents was selected. Young Ugandans aged 10–15 years were excluded given their reduced exposure to sexual activity and to SRH/HIV information as per national guidelines on sexuality education [20].

### Sampling

The first stage of sampling involved a random selection of 44 districts from the sampling frame of all districts in Uganda which were proportionately allocated to the 10 Uganda Bureau of Statistics (UBOS) statistical regions according to their total populations. Kampala was however purposively sampled due to its unique socioeconomic and demographic characteristics. The second stage involved selecting enumeration areas (EA) using the UBOS 2014 National Population and Housing Census sampling frame [21]. A total of 149 EAs (20 households per EA) were selected and allocated proportionately to the 44 sampled districts, so that districts with larger populations were allocated more EAs. The third stage involved assigning a number to each EA household and randomly selecting the ones to be interviewed by applying a numeric interval. A questionnaire was administered to all eligible and consenting persons aged 10–24 years found in the selected households.

### Measurements

For the present paper, measurements of interest are (a) family planning (FP) knowledge measured by awareness of at least one modern contraceptive method, (b) knowledge of sexually transmitted infections (STIs) measured by having ever heard about any infections that people can get from sexual intercourse, (c) comprehensive knowledge of HIV/AIDS measured by a composite indicator of awareness about HIV, knowledge about its transmission and correctly rejecting three out of five common misconceptions about HIV/AIDS, (d) self-reported perceived risk of being infected with STIs including HIV, and (e) self-reported perceived risk of becoming pregnant or impregnating a girl being rated as “at risk” versus “not at risk”. The outcome of interest being risky sexual behaviour was defined by a composite indicator of behaviour that increases one’s risk of contracting STIs (including HIV) and/or unintended pregnancies, namely being involved in sexual relations under the influence of alcohol or drugs, engaging in sexual activities without a condom, and having multiple sexual partners in the 6 months preceding the survey.

### Statistical analysis

Data was analysed using STATA 15. Exploratory analysis was conducted to provide descriptive statistics for adolescent’s socio-demographic characteristics. Logistic regression was conducted to determine the association between background characteristics of young people, their SRH/HIV knowledge, perceived SRH/HIV risk and being involved in risky sexual behaviour. Statistical analysis was conducted both at bivariate and multivariate level. All variables explored at the bivariate level were retained at multivariate level in order to document those with potential for correlation.

### Ethical considerations

Ethical approval for the study was obtained from Mildmay Uganda Research Ethics Committee and research approval from the Uganda National Council of Science and Technology. Due to the young age of the group targeted by the study and the sensitivity of the research topic, a number of ethical considerations related to the appropriateness of the research and the confidentiality, privacy and consent of the respondents arose.

The research questions and data collection tools used in the study were adapted from existing studies validated for use in the context where this study was conducted. In addition, they were discussed with relevant stakeholders and adapted to the existing national guidelines on age-appropriateness [20]. Potential risks related to the sensitivity of information provided by respondents were mitigated by the use of anonymous identifiers in form of study numbers on the questionnaires, thus de-linking the data collected from specific individuals. In addition, all respondents were interviewed individually in a private place within the homestead, ensuring parents/guardians did not listen in on the discussions. Finally, participation in the research was based on full informed consent of respondents, including verbal consent from parents/legal guardians for those less than 18 years of age.

## Results

### Background socio-demographic characteristics of respondents

A total of 2725 young people aged 15–24 years were interviewed, representing a 98% response rate. Background characteristics of the respondents by age group are given in Table 1. Majority in both age groups (81.8%) were from rural areas. Almost half (46.5%) of the 20–24 year old respondents were ever married or cohabitated, compared to 9.8% of the respondents aged 15–19 years. Moreover, 75.9% of 20–24 year old respondents were out of school, when compared to 28% of those aged 15–19 years. It was also observed that 10.9% of younger respondents were household heads or spouses. Finally, 17.7% of respondents across both age groups did not have a filial or marital relationship to the household head.

**Table 1:** Distribution of participants by background characteristics

Variable	15–19 years, *n* = 1647	20–24 years, *n* = 1078	15–24 years, *n* = 2725
	Freq. (%)	Freq. (%)	Freq. (%)
Residence			
Urban	261 (15.8)	236 (21.9)	497 (18.2)
Rural	1386 (84.2)	842 (78.1)	2228 (81.8)
Marital status			
Never married	1484 (90.2)	576 (53.5)	2060 (75.7)
Ever married	162 (9.8)	501 (46.5)	663 (24.3)
Gender			
Male	741 (45.0)	520 (48.2)	1261 (46.3)
Female	906 (55.0)	558 (51.8)	1464 (53.7)
Schooling status			
In school	1184 (72.0)	258 (24.1)	1442 (53.1)
Out of school	461 (28.0)	814 (75.9)	1275 (46.9)
Education level attained			
None	18 (1.1)	21 (2.0)	39 (1.4)
Some primary	833 (50.6)	422 (39.2)	1255 (46.1)
Secondary and above	794 (48.3)	633 (58.8)	1427 (52.4)
Relation to household head			
Head/spouse	179 (10.9)	458 (42.5)	637 (23.4)
Son/daughter	1165 (70.7)	441 (40.9)	1606 (59.0)
Other relative	303 (18.4)	178 (16.5)	481 (17.7)
Parents’ status			
Both parents alive	1181 (71.7)	650 (60.4)	1831 (67.2)
One parent alive	330 (20.0)	291 (27.0)	621 (22.8)
Both parents dead	136 (8.3)	135 (12.5)	271 (10.0)
Occupation			
Student	1003 (61.0)	203 (18.9)	1206 (44.4)
Peasant	263 (16.0)	369 (34.4)	632 (23.3)
Casual works	286 (17.4)	411 (38.3)	697 (25.7)
Unemployed	91 (5.5)	91 (8.5)	182 (6.7)
Region			
Central 1	198 (12.0)	128 (11.9)	326 (12.0)
Central 2	164 (10.0)	103 (9.6)	267 (9.8)
East Central	228 (13.8)	107 (9.9)	335 (12.3)
Eastern	218 (13.2)	156 (14.5)	374 (13.7)
Kampala	73 (4.4)	79 (7.3)	152 (5.6)
Karamoja	43 (2.6)	39 (3.6)	82 (3.0)
North	153 (9.3)	88 (8.2)	241 (8.8)
South West	255 (15.5)	185 (17.2)	440 (16.1)
West Nile	135 (8.2)	54 (5.0)	189 (6.9)
Western	180 (10.9)	139 (12.9)	319 (11.7)

### SRH/HIV knowledge, risk perception and risky sexual behaviour among respondents

Table 2 shows distribution of respondents’ SRH and HIV knowledge and risk indicators by age group. Overall, knowledge levels of FP, STIs and HIV across all young people were high (above 82%), with the older age group (20–24 years) displaying consistently higher levels of knowledge (7% higher on average) than the younger age group. Awareness of at least one modern FP method was high for both age groups, at an average of 94%. No major differences were observed between knowledge of STIs and comprehensive knowledge of HIV with a similar average of 85.6 and 86.2% across respondents for both indicators. Self-perceived risk of STIs (including HIV) and pregnancy among all respondents was high, with about half of all respondents reporting to feel at risk. For 20–24 year old respondents, self-reported perceived risk was consistently higher, with 61.5% feeling at risk of STIs compared to 46.2% of 15–19 year olds. Further, a combined proportion of 22.7% of respondents reported to have been involved in risky sexual behaviour exposing them to STI/HIV and/or pregnancy. Prevalence of risky sexual behaviour among the older group (34.6%) was more than double that of 15–19 year olds (14.9%).

**Table 2:** SRH/HIV knowledge and perceived risk by age group

Variable	15–19 years, *n* = 1647	20–24 years, *n* = 1078	15–24 years, *n* = 2725
	Freq. (%)	Freq. (%)	Freq. (%)
FP knowledge			
Yes	1499 (91.3)	1056 (98.0)	2555 (94.0)
No	142 (8.7)	22 (2.0)	164 (6.0)
Knowledge of STIs			
Yes	1326 (82.0)	964 (91.2)	2290 (85.6)
No	291 (18.0)	93 (8.8)	384 (14.4)
Knowledge of HIV/AIDS			
Yes	1379 (83.7)	970 (90.0)	2349 (86.2)
No	268 (16.3)	108 (10.0)	376 (13.8)
Perceived risk of STIs			
At risk	761 (46.2)	663 (61.5)	1424 (52.3)
Not at risk	886 (53.8)	415 (38.5)	1301 (47.7)
Perceived risk of pregnancy			
At risk	657 (39.9)	567 (52.6)	1224 (44.9)
Not at risk	990 (60.1)	511 (47.4)	1501 (55.1)
Risky sexual behaviour			
Yes	245 (14.9)	373 (34.6)	618 (22.7)
No	1402 (85.1)	705 (65.4)	2107 (77.3)

### Factors associated with risky sexual behaviour among young people

Being involved in risky sexual behaviour was regressed against background characteristics, SRH knowledge and self-perceived risk to SRH problems. Tables 3 and 4 show odds ratio estimates at bivariate and multivariate levels respectively for 15–19 and 20–24 year old respondents. For the 15–19 years group, factors associated with risky sexual behaviour after correcting for differences in age included being female versus male (aOR: 1.89, 95% CI: 1.37–2.61), being out of school versus being in school (aOR: 1.44, 95% CI: 0.87–2.41), having one parent alive versus having both (aOR: 1.56, 95% CI: 1.08–2.24), being involved in casual works versus being a student (aOR: 2.08, 95% CI: 1.27–3.39), being knowledgeable about FP methods (aOR: 3.46, 95% CI: 1.47–8.14), as well as the respondents’ self-perceived risk of being infected with STIs (aOR: 2.88, 95% CI: 1.72–4.82) (Table 3).

**Table 3:** Factors associated with having engaged in risky sexual behaviour among participants aged 15–19 years

Variable	Bivariate analysis				Multivariate analysis			
	OR	*p*-value	95% CI		aOR	*p*-value	95% CI	
Residence (ref: Rural)	0.80	0.27	0.54	1.19	0.80	0.41	0.48	1.35
Marital Status (ref: Ever married)	0.38	< 0.001	0.26	0.55	0.98	0.95	0.57	1.71
Age (Continuous)	1.60	< 0.001	1.44	1.78	1.44	< 0.001	1.26	1.64
Gender (ref: Female)	1.68	< 0.001	1.27	2.20	1.89	< 0.001	1.37	2.61
Schooling Status (ref: In school)	2.59	< 0.001	1.96	3.42	1.44	0.16	0.87	2.41
Education level (ref: Secondary +)								
None	2.16	0.15	0.76	6.17	1.59	0.48	0.44	5.70
Some Primary	0.95	0.69	0.72	1.24	0.89	0.55	0.62	1.29
Relation to household head (ref: Child)								
Head/spouse	2.00	< 0.001	1.35	2.94	1.13	0.64	0.67	1.92
other relative	1.42	0.04	1.01	2.00	1.14	0.55	0.74	1.75
Parents’ Status (ref: Both alive)								
Both parents dead	1.54	0.07	0.96	2.45	1.20	0.52	0.69	2.12
One parent alive	1.80	< 0.001	1.32	2.47	1.56	0.02	1.08	2.24
Occupation (ref: Student)								
Peasant	2.42	< 0.001	1.69	3.46	1.11	0.70	0.65	1.91
Casual work	2.99	< 0.001	2.14	4.19	2.08	< 0.001	1.27	3.39
Unemployed	1.20	0.59	0.62	2.33	0.90	0.79	0.40	1.99
Region (ref: Central 1)								
Central 2	1.74	0.08	0.94	3.19	2.04	0.04	1.04	4.00
East Central	0.95	0.86	0.51	1.77	1.42	0.32	0.71	2.82
Eastern	1.19	0.57	0.65	2.18	1.40	0.33	0.72	2.72
Kampala	0.89	0.81	0.36	2.20	1.27	0.67	0.43	3.78
Karamoja	2.55	0.03	1.10	5.91	2.49	0.08	0.91	6.78
North	1.49	0.22	0.79	2.81	1.49	0.29	0.72	3.09
South West	1.39	0.27	0.78	2.46	1.31	0.41	0.69	2.50
West Nile	1.82	0.06	0.97	3.43	2.35	0.02	1.17	4.73
Western	2.89	< 0.001	1.65	5.08	3.31	< 0.001	1.77	6.18
SRH Knowledge and Perceived Risk								
FP knowledge (ref: No)	3.64	< 0.001	1.68	7.88	3.46	< 0.001	1.47	8.14
Knowledge of STIs (ref: No)	1.87	< 0.001	1.23	2.85	1.41	0.16	0.87	2.29
Knowledge of HIV/AIDS (ref: No)	0.96	0.83	0.67	1.38	0.93	0.73	0.61	1.42
Perceived risk of STIs (ref: None)	3.25	< 0.001	2.42	4.36	2.88	< 0.001	1.72	4.82
Perceived risk of pregnancy (ref: None)	2.52	< 0.001	1.91	3.32	1.01	0.96	0.62	1.66

**Table 4:** Factors associated with having engaged in risky sexual behaviour among participants aged 20–24 years

Variable	Bivariate analysis				Multivariate analysis			
	OR	*p*-value	95% CI		aOR	*p*-value	95% CI	
Residence (ref: Rural)	1.34	0.06	0.99	1.80	1.52	0.04	1.03	2.24
Marital Status (ref: Ever married)	0.74	0.02	0.57	0.95	0.87	0.44	0.60	1.25
Age (Continuous)	1.17	< 0.001	1.08	1.27	1.12	0.02	1.02	1.23
Gender (ref: Female)	1.67	< 0.001	1.30	2.15	2.10	< 0.001	1.56	2.81
Schooling Status (ref: In school)	1.89	< 0.001	1.38	2.60	1.27	0.42	0.71	2.28
Education level (ref: Secondary +)								
None	1.58	0.31	0.65	3.80	1.97	0.17	0.74	5.22
Some Primary	1.28	0.06	0.99	1.66	1.46	0.02	1.07	2.01
Relation to household head (ref: Child)								
Head/spouse	1.64	< 0.001	1.24	2.17	1.29	0.17	0.90	1.87
Other relative	1.62	0.01	1.12	2.33	1.55	0.04	1.03	2.34
Parents’ Status (ref: Both alive)								
Both parents dead	1.39	0.09	0.95	2.03	1.11	0.62	0.73	1.70
One parent alive	1.11	0.49	0.83	1.48	0.97	0.87	0.71	1.34
Occupation (ref: Student)								
Peasant	1.73	0.01	1.17	2.56	1.03	0.93	0.52	2.04
Casual works	2.56	< 0.001	1.74	3.75	1.48	0.23	0.78	2.79
Unemployed	1.44	0.20	0.82	2.51	1.01	0.97	0.47	2.19
Region (ref: Central 1)								
Central 2	1.31	0.32	0.77	2.25	1.31	0.37	0.72	2.36
East Central	1.39	0.23	0.82	2.35	1.42	0.23	0.80	2.53
Eastern	0.87	0.60	0.53	1.44	0.95	0.84	0.56	1.62
Kampala	1.05	0.88	0.58	1.89	0.60	0.16	0.30	1.22
Karamoja	0.85	0.68	0.39	1.84	0.79	0.60	0.33	1.90
North	0.76	0.36	0.42	1.37	0.72	0.31	0.37	1.37
South West	0.81	0.39	0.50	1.31	0.72	0.23	0.42	1.22
West Nile	1.21	0.56	0.63	2.34	1.06	0.87	0.53	2.13
Western	1.14	0.61	0.69	1.88	1.13	0.65	0.66	1.96
SRH Knowledge and Perceived Risk								
FP knowledge (ref: No)	1.82	0.24	0.67	4.97	1.40	0.53	0.49	4.05
Knowledge of STIs (ref: No)	1.11	0.64	0.71	1.75	1.07	0.78	0.65	1.76
Knowledge of HIV/AIDS (ref: No)	1.42	0.12	0.91	2.21	1.55	0.08	0.95	2.53
Perceived risk of STIs (ref: None)	1.27	0.07	0.98	1.65	0.84	0.45	0.53	1.32
Perceived risk of pregnancy (ref: None)	1.32	0.03	1.03	1.70	1.56	0.05	1.01	2.43

For the 20–24 years old respondents, significant predictors of risky sexual behaviour included living in a rural area (aOR: 1.52, 95% CI: 1.03–2.24), being female versus male (aOR: 2.10, 95% CI: 1.56–2.81), having some primary versus secondary education (aOR: 1.46, 95% CI: 1.07–2.01), not having a filial or marital relationship with the head of household (aOR: 1.55, 95% CI: 1.03–2.34), and self-perceived risk of pregnancy (aOR: 1.56, 95% CI: 1.01–2.43) (Table 4).

## Discussion

In a context where sexually transmitted infections and teenage pregnancies are still a major public health problem, and where HIV prevalence seems to be rebounding, it is important to understand the emerging drivers of the epidemic and the opportunities for closer SRH/HIV integration in prevention services. This study goes beyond descriptive statistics about SRH indicators in young people to provide an incisive analysis of the connection between the knowledge – risk perception – practices continuum. Unpacking this important nexus can help to identify likely sources of stagnation along the continuum.

The high rates of SRH and HIV knowledge observed among respondents confirm our assumption that increasing HIV prevalence among young people and stagnating teenage pregnancy prevalence in Uganda are not primarily due to lack of information among this group. Both younger and older respondents in our study displayed levels of knowledge above 82%, though as expected the 20–24 year olds had higher SRH awareness across all surveyed areas. However, the disparity of up to 9 percentage points in average SRH/HIV knowledge levels among respondents (from 94% average knowledge about FP to 86% average knowledge about HIV) points to a missed opportunity to further integrate SRH/HIV information and educational campaigns for young people in Uganda, while keeping in mind that SRH/HIV prevention interventions focused exclusively on providing information have been shown to link weakly to risk behaviour reductions [22–25]. Our results also point to the need of repurposing the delivery of SRH/HIV messages in primary care.

Whereas SRH knowledge among respondents is high, our analysis shows it is also a significant predictor of risky sexual behaviour. Contrary to what could be expected, more knowledgeable young people seem also to be more likely to have engaged in risky sexual behaviour. This is particularly the case for FP knowledge among 15–19 year olds, which might be indicative of this young group’s tendency to source SRH information from their existing sexual networks as they experiment and gain more experience. The same variable is not a significant predictor of risky sexual behaviour among the older group (20–24 year olds), which face a different set of factors associated with risky sexual behaviour.

Overall, our findings on knowledge levels and individual behaviour support other studies that claim protective FP/STI/HIV knowledge alone does not necessarily result in safe-sex behaviour [26, 27]. In particular, risky contraceptive use behaviour observed among young people with significant SRH knowledge levels might be partially attributed to negative norms, fears and attitudes that affect their uptake of condoms and other contraceptives, as well as to challenges accessing services [28–31]. Closer integration of FP and HIV counselling and testing services for young people is therefore a pending deliverable and an opportunity to improve contraceptive uptake among young Ugandans displaying risk sexual behaviours that expose them to HIV [32].

Nearly 1 in 2 young respondents reported feeling at risk of SRH problems, with perceived risk of STIs (including HIV) featuring consistently higher (average 52%) than pregnancy risk (average 45%) across both younger and older respondents. While the perceived risk trend was seen to increase with age, these results should be interpreted in light of the tendency of younger people to overestimate risk [33].

The high levels of self-perceived vulnerability reported by respondents would be expected to contribute to a firm personal motivation to reduce risk exposure. However, our research findings indicate the contrary: Both self-perceived risk of STIs (including HIV) among the younger group and vulnerability to pregnancy among the older group are associated with risky instead of protective sexual behaviour. This is not an unusual finding, attributed by Millestein et al. to the type of measure used (nonconditional) and the cross-sectional study design [33]. Our results might therefore indicate that self-perceived vulnerability is a reflection of risky sexual behaviour, although causality cannot be established. Personal motivation to reduce sexual risk behaviour might also be influenced by social factors and perceived social norms [34–37], which were variables not included in our analysis.

Our research supports the findings from other studies [37, 38] that certain social networks and institutions might have a protective role against risky sexual behaviour by young people. We found in particular that school enrolment among the age group 15–19 seems to have a protective effect on sexual behaviour, while doing casual work instead of studying was associated with increased sexual risk behaviours among this age group. These results support the conclusions from prior studies. Research by Hargreaves et al. found that among unmarried and rural young South Africans aged 14–25 years, school attendance was protective due to the structure of sexual networks associated with lower-risk sexual behaviours [39], while Behrman and De Neve et al. highlighted completion of primary and secondary education as having positive effects on exposure to sexual activity and reduction in the cumulative risk of HIV infection [40, 41]. Educational achievement among the 20–24 year olds was also found to have a protective effect on sexual behaviour, which supports findings from literature pointing at decreased HIV prevalence and less risky sexual behaviour among the more educated [42, 43].

Another important social institution with direct effect over young people’s risky sexual behaviour is the family [44]. Our results indicate that single orphanhood among the younger respondent group and lack of a filial or marital relationship to the head of household among the older group were significant variables affecting risky sexual behaviour. This is supported by other research that points at limited perceived parental monitoring and orphanhood (both single and double) to be associated with poorer health behaviours [45, 46].

Our findings reaffirm existing programming recommendations for SRH/HIV prevention, including the need to focus not only on individual-level behavioural interventions, but also on social and structural factors and institutions that affect individual behaviour [47]. Our research also emphasizes that certain demographic groups (young women and rural residents) seem to also be more vulnerable to higher risk sexual practices, which calls for SRH/HIV prevention programmes to further target these vulnerable groups.

While this study contributes to the existing evidence on knowledge and risk perceptions, and their association with risky sexual behaviour, the results presented should be examined taking into account a few study limitations. These include measurement of various socio-behavioural variables on the basis of composite indicators such as SRH knowledge, challenges in adequately assessing attitudes and perceptions (e.g. risk perception), as well as reliance on self-reports for sensitive questions related to risky sexual behaviour which could lead to underestimation of its prevalence. To mitigate these challenge however, we used tools that have already been tested and validated for use in the context where this study was conducted. In addition, while we acknowledge that there are multiple social and structural factors that influence individual risky sexual behaviour (social norms, service accessibility and quality, economic pressures), our study did not explore in depth these reasons. Finally, the cross-sectional nature of the research inhibits our ability to establish cause and effect for certain indicators of interest.

## Conclusions

Our study found that although respondent levels of knowledge about FP, STIs and HIV were high, young people also had a high self-perceived SRH/HIV risk and displayed significant levels of risky sexual behaviour. These trends were significantly higher among the 20–24 age group than among 15–19 year olds. This seems to point at a lack of functional skills among young people to avert SRH/HIV related risks.

We also found that being female, being out of school or involved in casual work, being a single orphan, being knowledgeable about FP, and perceiving oneself to be at risk of acquiring an STI/HIV were significant predictors of risky sexual behaviour among 15–19 year olds. Associated factors particular to the 20–24 year old group included being resident in rural areas, not having reached secondary education, not having a filial or marital relationship with the head of household, and perceiving oneself to be at risk of an unwanted pregnancy. Our findings contribute to the available evidence that risky sexual sexual behaviour is not only a function of adequate disease prevention knowledge, but also of young people’s ability to engage in preventive behaviour [48], of their positive engagement in surrounding social structures (school and family) and of their ability to negotiate existing social norms.

Given that one in five young Ugandans are engaged in risky sexual behaviour, there is a clear need to scale up SRH/HIV prevention programs that adopt a holistic approach to reduction of risky sexual behaviour. This must be done by combining individual level interventions through information, risk awareness, skill development and discussion of cost-benefit calculations involved in the adoption of SRH/HIV preventive behaviours with interventions targeting social structures affecting individual behaviour.

A French translation of this article has been included as Additional file 1.

A Portuguese translation of the abstract has been included as Additional file 2.

## Additional files

Additional file 1: Translation of this article into French. (PDF 266 kb)

Additional file 2: Translation of the abstract of this article into Portuguese. (PDF 271 kb)

## Abbreviations

EA: Enumeration Area
FP: Family Planning
HIV: Human Immunodeficiency Virus
SDG: Sustainable Development Goal
SRH: Sexual and Reproductive Health
STI: Sexually Transmitted Infection
UBOS: Uganda Bureau of Statistics
