# Supplementary material for: The knowledge-risk-behaviour continuum among young Ugandans: what it tells us about SRH/HIV integration
Source: BMC Public Health. 2019 May 29;19(Suppl 1):604. doi: 10.1186/s12889-019-6809-y (PMC6538546; doi:10.1186/s12889-019-6809-y)
Supplement: Supplementary file 1 — Translation of this article into French. (PDF 266 kb) [file 12889_2019_6809_MOESM1_ESM.pdf]

Le continuum connaissances/risque/comportement parmi les jeunes Ougandais : Ce qu'il nous dit de l'intégration des services de SSR/anti-VIH

Raquel Palomino González[1,] Damazo Twebaze Kadengye[2,5*] , Roy William Mayega[3, 4]

[1] United Nations Population Fund (UNFPA), Kampala, Uganda, ragonzalez@unfpa.org
[2] African Population and Health Research Center, Nairobi, Kenya, dkadengye@aphrc.org
[3] School of Public Health, Makerere University, Kampala, Uganda, rmayega@musph.ac.ug
[4] AH Consulting, Kampala, Uganda
[5] D.T.K. contribution to this paper occurred while he worked with the School of Statistics and Planning, Makerere University; Kampala, Uganda and AH Consulting Ltd., Kampala, Uganda.
Autor para correspondência:

* Auteur pour la correspondence: Kadengye D.T., African Population and Health Research Center, Manga Cl, Nairobi, Kenya, E-mail: dkadengye@gmail.com / dkadengye@aphrc.org

RÉSUMÉ

Contexte : Avec une prévalence du virus de l'immunodéficience humaine (VIH) de 2,1 % parmi les jeunes de 15 à 24 ans, il existe des opportunités d'intégration plus poussée des services de santé sexuelle et reproductive (SSR) et de prévention du VIH pour les jeunes en Ouganda. Nous examinons un éventail de facteurs qui contribuent à la variance dans les comportements sexuels à risque des jeunes Ougandais.

Méthodes : Une étude transversale des foyers, représentative à l'échelon national, a été conduite entre février et mars 2016. Le questionnaire utilisé a évalué les connaissances, les attitudes et les pratiques liées à la SSR parmi les jeunes âgés de 10 à 24 ans. Un indicateur composé a été créé pour évaluer les comportements sexuels à risque, définis comme l'implication dans des relations sexuelles sous l'emprise de l'alcool ou de drogues ou dans des activités sexuelles sans préservatif, et le fait d'avoir eu des partenaires sexuels multiples dans les 6 mois précédant l'enquête. L'analyse d'exploration a été conduite afin de fournir des

statistiques descriptives. Une régression logistique a été conduite pour déterminer les facteurs associés à un comportement sexuel à risque. Cette analyse se concentre sur la sous-catégorie des 15-24 ans, qui comprend 2 725 participants.

Résultats : Le niveau des connaissances parmi les participants du planning familial (PF), des infections sexuellement transmissibles (IST) et du VIH était élevé (plus de 82 %). Le risque perçu pour soi déclaré d'IST et de grossesse était plus élevé parmi les participants âgés de 20-24 ans, avec 61,5 % se sentant à risque d'IST comparé à 46,2 % des 15-19 ans. Au total, 22,7 % des participants ont rapporté avoir eu un comportement sexuel à risque. Les facteurs associés à un comportement sexuel à risque parmi le groupe des 15-19 ans incluaient le genre, le fait d'être orphelin d'un parent, le travail occasionnel, le statut scolaire, la connaissance du PF et le risque perçu pour soi des IST/du VIH. Pour les participants âgés de 20-24 ans, les facteurs associés significatifs incluaient le genre, le niveau d'éducation, la relation avec le chef de famille, le lieu de résidence et le risque perçu pour soi de grossesse.

Conclusions : Malgré un niveau de connaissances général élevé de la SSR/du VIH et du risque perçu de VIH, les comportements sexuels à risque parmi les jeunes Ougandais restent très présents. Les lacunes dans l'efficacité de la réponse intégrée à la SSR/au VIH pour les jeunes doivent être traitées et des interventions ciblées concentrées sur la prévention holistique au niveau individuel par l'information, la conscience des risques et le développement des compétences devraient être combinées à des interventions ciblant les structures sociales affectant le comportement individuel.

Mots clés : SSR, VIH, connaissances, perception des risques, comportement sexuel, jeunes, Ouganda

CONTEXTE

Les bénéfices possibles dérivés des associations entre SSR et VIH au niveau des politiques, des systèmes et de la fourniture de services sont largement reconnus aujourd'hui [1-6]. L'accent mis à nouveau sur le programme d'intégration de la SSR/du VIH, qui cherche à refaçonner la fourniture de services de santé pour les services de SSR et de lutte contre le VIH en fournissant des services de santé complets et des références dans ce domaine [7], peut efficacement accélérer l'atteinte des objectifs de 90-90-90 [8] et faire avancer vers l'objectif de développement durable (ODD) 3 qui est de mettre fin à l'épidémie de SIDA d'ici 2030 [9].

Pour les jeunes en particulier, l'intégration des services de SSR/anti-VIH doit faire monter le recours aux services de SSR et de lutte contre le VIH, améliorer la connaissance du statut VIH, promouvoir les relations sexuelles protégées, réduire la stigmatisation et la discrimination liées au VIH, apporter une meilleure expérience des clients et une plus grande satisfaction, mieux contribuer à une double protection, ainsi qu'à une compréhension et une protection accrues des droits individuels [1,10]. En Ouganda, l'intégration de la SSR/du VIH a eu lieu au niveau des politiques, des systèmes et de la fourniture de services depuis 2012, lorsque le Programme de contrôle du sida et la Division de la santé reproductive du ministère de la santé ougandais ont été mandatés pour les mettre à l'ordre du jour [11]. L'intégration de la SSR et du VIH dans tous les services de soins et de traitement constituait un objectif stratégique du Plan stratégique

national pour le VIH&SIDA 2011/12 -2014/15 [12], et, depuis lors, elle a lieu dans la plupart des politiques, stratégies et orientations concernant la SSR et le VIH dans le pays [13-14].

L'efficacité de la réponse intégrée à la SSR/au VIH obtenue à l'échelle nationale peut être mesurée avec la liste des indicateurs mis au point par le Groupe de travail interinstitutions sur les liens entre la SSR et le VIH, à travers 8 secteurs différents. Les indicateurs incluent, entre autres, le pourcentage d'adultes âgés de 15 à 49 ans qui ont eu plus d'un partenaire sexuel au cours des 12 derniers mois et rapportent l'utilisation d'un préservatif lors du dernier rapport sexuel, et le taux de prévalence du VIH parmi les jeunes [15]. Si l'on considère les performances de l'Ouganda sur ces indicateurs, et malgré l'engagement du gouvernement à accélérer le déploiement de cet ordre du jour aux niveaux national et local, des lacunes significatives persistent concernant les résultats de SSR/VIH parmi les jeunes.

On estime que 67 jeunes Ougandais contractent le VIH chaque jour. Cela représente 44,1 % de toutes les nouvelles infections dans le pays, dont la majorité est d'origine sexuelle [14]. Le taux de prévalence du VIH parmi les jeunes de 15 à 24 ans est de 2,1 % [16]. Les jeunes femmes, en particulier, ont un risque significatif à la fois de grossesse non désirée et d'infection au VIH. La prévalence du VIH parmi les 15-24 ans est 4 fois plus élevée parmi les femmes (3,3 %) que parmi les hommes (0,8 %) [16], et 23,5 % des jeunes filles de 15 à 19 ans ont déjà accouché [17].

Il existe toujours des raisons importantes de pousser l'intégration des services de SSR/de lutte contre le VIH pour les jeunes en Ouganda [18]. Permettre aux jeunes Ougandais de réduire leur exposition au risque du VIH constitue un objectif fondamental de l'intégration de la SSR et du

VIH, et une compréhension plus détaillée des facteurs clés contribuant à la variance des comportements à risque liés à la SSR/au VIH parmi les jeunes Ougandais peut aider à concevoir des interventions qui réduisent efficacement l'exposition au risque du VIH parmi ce groupe cible. L'objectif de cette étude était donc d'établir les niveaux de connaissances, les attitudes et les pratiques en lien avec la santé sexuelle et reproductive des jeunes Ougandais.

MÉTHODES

Conception de l'étude

Une étude transversale des foyers a été conduite en Ouganda entre février et mars 2016. L'étude ciblait un échantillon de foyers représentatif au niveau national, sur la base des recommandations pratiques du Département de l'ONU des Affaires économiques et sociales, dans l'établissement d'échantillons d'enquête sur les foyers [19]. La taille totale de l'échantillon englobait 2 976 foyers. Les calculs sur la taille de l'échantillon ont été basés sur les hypothèses suivantes : niveau de confiance 95 %, pourcentage de jeunes âgés de 10 à 24 ans ayant des connaissances complètes sur le VIH/SIDA établi à 40 %, effet du plan d'échantillonnage de 2,0, taux de non-réponse de 20 %, marge d'erreur/de précision de 10 % et moyenne estimée de 1,48 répondant par foyer âgé de 10 à 24 ans.

Étant donné les questions de recherche sélectionnées pour ce papier qui se concentrent sur l'activité sexuelle et sur les connaissances de la SSR/du VIH, c'est la sous-catégorie de l'étude des 15-24 ans comprenant 2 725 répondants qui a été sélectionnée. Les jeunes de 10 à 15 ans ont été exclus en raison de leur activité sexuelle et de leur exposition aux informations sur la

SSR/le VIH réduites, comme stipulé par les recommandations nationales sur l'éducation à la sexualité [20].

Échantillonnage

La première étape d'échantillonnage impliquait une sélection aléatoire de 44 districts, parmi le cadre d'échantillonnage de tous les districts en Ouganda, qui ont été attribués de manière proportionnelle aux 10 régions statistiques constituant le Bureau des statistiques de l'Ouganda (UBOS) en fonction de leurs populations totales. Toutefois, Kampala a été inclus d'office en raison de ses caractéristiques socio-économiques et démographiques uniques. La deuxième étape impliquait la sélection de zones de dénombrement (ZD) en utilisant le cadre d'échantillonnage du recensement national de la population et du logement de 2014 de l'UBOS [21]. Au total, 149 ZD (20 foyers par ZD) ont été sélectionnées et attribuées de manière proportionnelle aux 44 districts, de manière que les districts plus peuplés ont reçu aussi plus de ZD. La troisième étape impliquait d'affecter un nombre à chaque foyer de ZD et à sélectionner au hasard ceux à interroger en appliquant un intervalle numérique. Un questionnaire a été soumis à toutes les personnes éligibles et consentantes âgées de 10 à 24 ans localisées dans les foyers sélectionnés.

Mesures

Pour le présent papier, on s'attache à mesurer (a) la connaissance du planning familial (PF) mesurée par la connaissance d'au moins une méthode contraceptive moderne, (b) la connaissance des infections sexuellement transmissibles (IST) mesurée par le fait d'avoir entendu parler des infections que l'on peut contracter au cours de rapports sexuels, (c) la

connaissance complète du VIH/SIDA mesurée par un indicateur composé de la connaissance du VIH, de la connaissance des modes de transmission et par le rejet correct de trois des cinq idées reçues courantes sur le VIH/SIDA, (d) le risque perçu pour soi rapporté d'une IST, y compris du VIH, et (e) le risque perçu pour soi rapporté d'une grossesse ou de féconder une fille considérée comme « à risque » ou « pas à risque ». Le résultat d'intérêt étant les comportements sexuels à risque, il a été défini par un indicateur composé du comportement qui augmente le risque de contracter des IST (VIH inclus) et/ou des grossesses non désirées, à savoir l'implication dans des rapports sexuels sous l'emprise de l'alcool ou de drogues ou dans des activités sexuelles sans préservatif, et le fait d'avoir eu des partenaires sexuels multiples dans les 6 mois précédant l'enquête.

Analyse statistique

Les données ont été analysées avec le logiciel STATA 15. L'analyse d'exploration a été conduite afin de fournir des statistiques descriptives sur les caractéristiques socio-démographiques des adolescents. Une régression logistique a été conduite pour déterminer le lien entre les caractéristiques de contexte des jeunes, leur connaissance de la SSR/du VIH, le risque perçu de SSR/VIH et l'implication dans les comportements sexuels à risque. L'analyse statistique a été menée à la fois sur les niveaux à deux variables et à variables multiples. Toutes les variables explorées au niveau des deux variables ont été retenues au niveau des variables multiples, afin de documenter celles avec un potentiel de corrélation.

Considérations éthiques

L'approbation éthique de l'étude a été obtenue auprès du Comité d'éthique Mildmay des recherches de l'Ouganda et l'approbation de la recherche auprès du Conseil national de la science et de la technologie de l'Ouganda. En raison du jeune âge du groupe ciblé par l'enquête et de la sensibilité du sujet de la recherche, de nombreuses considérations éthiques liées à l'adéquation de la recherche, la confidentialité, le respect de la vie privée et le consentement des participants sont apparues.

Les questions de la recherche et les outils de collecte de données utilisés au cours de l'étude ont été adaptés à partir d'études existantes validées pour une utilisation dans le contexte où cette enquête a été conduite. De plus, ils ont été débattus avec les parties prenantes et ajustés aux directives nationales existantes quant à leur adéquation à l'âge des répondants [20]. Les risques potentiels liés à la sensibilité des informations fournies par les répondants ont été réduits en utilisant des identifiants anonymes sous forme de numéros d'étude sur les questionnaires, qui ne permettaient pas de relier les données collectées à des individus spécifiques. En outre, tous les répondants ont été interrogés individuellement dans un lieu confidentiel au sein du foyer, en s'assurant que les parents/tuteurs n'écoutaient pas les discussions. Enfin, la participation à la recherche a été basée sur le consentement éclairé des répondants, avec l'accord oral des parents/tuteurs pour les mineurs.

RÉSULTATS

Caractéristiques socio-démographiques des répondants

Au total, 2 725 jeunes âgés de 15 à 24 ans ont été interrogés, ce qui représente un taux de réponses de 98 %. Les caractéristiques de contexte des répondants par groupe d'âges sont données dans le Tableau 1. La majorité dans les deux groupes d'âges (81,8 %) provenait de zones rurales. Presque la moitié (46,5 %) des 20-24 ans avaient déjà été mariés ou en concubinage, comparé à 9,8 % des répondants âgés de 15 à 19 ans. Par ailleurs, 75,9 % des répondants des 20-24 ans n'étaient plus scolarisés, comparé à 28 % des 15-19 ans. On a également observé que 10,9 % des répondants les plus jeunes étaient chefs de famille ou conjointes. Enfin, 17,7 % des répondants au sein des deux groupes d'âges n'avaient pas de lien filial ou marital avec le chef de famille.

Connaissance de la SSR/du VIH, perception du risque et comportements sexuels à risque parmi les répondants

Le Tableau 2 donne la répartition de la connaissance de la SSR/du VIH parmi les répondants et les indicateurs de risque par groupe d'âges. Au total, les niveaux de connaissances du PF, des IST et du VIH étaient élevés (plus de 82 %) parmi tous les jeunes, avec des niveaux de connaissances toujours plus élevés (en moyenne de 7 %) chez le groupe des plus âgés (20-24 ans). La connaissance d'au moins une méthode moderne de PF était élevée pour les deux groupes d'âges, avec une moyenne de 94 %. Aucune différence notable n'a été observée entre la connaissance des IST et une connaissance complète du VIH, avec des moyennes de 85,6 % et 86,2 % pour les deux indicateurs. Le risque d'IST perçu pour soi (y compris du VIH) ou de grossesse parmi tous les répondants était élevé, avec environ la moitié rapportant se sentir à risque. Pour les 20-24 ans, le risque perçu pour soi était toujours plus élevé, avec 61,5 % se

déclarant à risque d'IST, comparé à 46,2 % pour les 15-19 ans. En outre, une proportion combinée de 22,7 % des répondants a déclaré avoir été impliqués dans une conduite à risque les exposant aux IST/au VIH et/ou à une grossesse. La prévalence des comportements sexuels à risque parmi le groupe plus âgé (34,6 %) était le double de celle des 15-19 ans (14,9 %).

Facteurs associés à des comportements sexuels à risque parmi les jeunes

L'implication dans des comportements sexuels à risque a été analysée à l'aune des caractéristiques de contexte, des connaissances sur la SSR et du risque perçu pour soi de problèmes de SSR. Les Tableaux 3 et 4 indiquent les rapports de cote estimés au niveau des bivariables et multivariables, respectivement pour les répondants âgés de 15-19 ans et ceux âgés de 20-24 ans. Pour le groupe des 15-19 ans, les facteurs associés à un comportement sexuel à risque après correction selon l'âge incluent être une femme plutôt qu'un homme (RCc : 1,89, IC 95 % : 1,37 – 2,61), être déscolarisé plutôt que scolarisé (RCc : 1,44, IC 95 % : 0,87 – 2,41), avoir un parent en vie plutôt que deux (RCc : 1,56, IC 95 % : 1,08 – 2,24), travailler occasionnellement plutôt qu'être étudiant (RCc : 2,08, IC 95 % : 1,27 – 3,39), être au courant des méthodes de PF (RCc : 3,46, IC 95 % : 1,47 – 8,14), ainsi que le risque perçu pour soi d'être infecté par des IST (RCc : 2,88, IC 95 % : 1,72 – 4,82) (Tableau 3).

Pour les répondants âgés de 20-24 ans, les indicateurs significatifs de comportements sexuels à risque incluent de vivre en zone rurale (RCc : 1,52, IC 95 % : 1,03 – 2,24), d'être une femme plutôt qu'un homme (RCc : 2,10, IC 95 % : 1,56 - 2,81), d'avoir une éducation secondaire plutôt que primaire (RCc : 1,46, IC 95 % : 1,07 - 2,01), de ne pas avoir de lien filial ou marital avec le

chef de famille (RCc : 1,55, IC 95 % : 1,03 - 2,34), et de percevoir un risque pour soi de grossesse (RCc : 1,56, IC 95 % : 1,01 – 2,43) (Tableau 4).

DISCUSSION

Dans un contexte où les infections sexuellement transmissibles et les grossesses d'adolescentes constituent encore un problème de santé public majeur et où la prévalence du VIH semble connaître un rebond, il est important de comprendre les moteurs émergents de l'épidémie et les opportunités d'une intégration plus marquée de la SSR/du VIH dans les services de prévention. Cette étude va au-delà des statistiques descriptives sur les indicateurs de SSR parmi les jeunes, afin de fournir une analyse incisive du lien entre les connaissances/la perception des risques/les pratiques. Démêler ce nœud important peut permettre d'identifier les sources probables de stagnation le long de ce continuum.

Les taux élevés de connaissance de la SSR et du VIH constatés parmi les répondants confirment notre supposition que l'augmentation de la prévalence du VIH parmi les jeunes et la stagnation de la prévalence des grossesses chez les adolescentes en Ouganda ne sont pas dues principalement à un manque d'informations au sein de ce groupe. Les répondants les plus jeunes et les plus âgées de notre étude affichent des niveaux de connaissance supérieurs à 82 %, même si, comme on peut s'y attendre, les 20-24 ans ont de meilleures connaissances de la SSR dans tous les secteurs étudiés. Cependant, les disparités qui peuvent atteindre 9 points dans les niveaux moyens de connaissance de la SSR/du VIH parmi les répondants (de 94 % concernant le PF à 86 % concernant le VIH) indiquent une occasion manquée de mieux intégrer des informations de SSR/VIH et des campagnes d'éducation visant les jeunes en Ouganda, tout

en gardant à l'esprit que les interventions de prévention sur la SSR/le VIH ciblant exclusivement la fourniture d'informations sont faiblement liées à une réduction des comportements à risque [22-25]. Nos résultats indiquent également le besoin de repenser la façon de délivrer des messages sur la SSR/le VIH en soins primaires.

Alors que la connaissance de la SSR est élevée parmi les répondants, notre analyse indique qu'elle est également un indicateur significatif de comportements sexuels à risque. Contrairement aux attentes, plus les jeunes ont de connaissances, plus ils semblent susceptibles d'avoir eu des comportements sexuels à risque. C'est particulièrement le cas des connaissances de PF parmi les 15-19 ans, qui semblent indiquer la tendance au sein de ce groupe d'obtenir ses informations de SSR auprès de leurs réseaux sexuels, à mesure qu'ils expérimentent et gagnent en expérience. La même variable n'est pas un indicateur significatif de comportements sexuels à risque parmi le groupe plus âgé (20-24 ans), qui fait face à un ensemble différent de facteurs associés à des comportements sexuels à risque.

Globalement, nos découvertes sur les niveaux de connaissances et les comportements individuels renforcent d'autres études qui affirment que les connaissances des PF/IST/VIH seules n'entraînent pas nécessairement des pratiques sexuelles sans risque [26-27]. En particulier, les comportements de contraception risqués observés parmi les jeunes avec des niveaux élevés de connaissances de la SSR pourraient être attribués à des normes négatives, des craintes et des attitudes qui affectent leur utilisation des préservatifs et autres contraceptifs, ainsi qu'à des obstacles d'accès aux services [28-31]. Une intégration plus étroite des services de conseil et de dépistage pour le PF et la prévention du VIH auprès des jeunes est

donc un impératif et constitue une occasion d'améliorer l'adoption de contraceptifs parmi les jeunes Ougandais présentant des comportements sexuels à risque qui les exposent au VIH [32].

Presque 1 jeune répondant sur 2 a déclaré se sentir à risque de problèmes de SSR avec un risque perçu d'IST (y compris le VIH) toujours plus élevé (moyenne 52 %) que le risque de grossesse (moyenne 45 %) parmi les répondants à la fois les plus jeunes et les plus âgés. Alors que la tendance du risque perçu augmente avec l'âge, ces résultats doivent être interprétés à l'aune de la tendance des gens jeunes à surestimer le risque [33].

Les niveaux élevés de vulnérabilité perçue pour soi déclarée par les répondants devraient contribuer à une forte motivation personnelle à réduire son exposition aux risques. Cependant, les résultats de la recherche indiquent le contraire : Le risque d'IST (y compris VIH) perçu pour soi parmi le groupe plus jeune et la vulnérabilité à la grossesse parmi le groupe plus âgé sont associés à des comportements sexuels risqués plutôt que protégés. Ce résultat n'est pas inhabituel, il est attribué par Millestein et coll. au type de mesure utilisé (non conditionné) et à la conception de l'étude transversale [33]. Par conséquent, nos résultats peuvent indiquer que la vulnérabilité perçue pour soi reflète un comportement sexuel à risque, bien qu'il soit impossible d'établir la causalité. La motivation personnelle de réduire son comportement sexuel à risque peut aussi être influencée par des facteurs sociaux et des normes sociales perçues [34-37], des variables qui n'ont pas été incluses dans notre analyse.

Notre recherche confirme les résultats d'autres études [37-38] indiquant que certains réseaux sociaux et certaines institutions peuvent jouer un rôle protecteur contre les comportements sexuels à risque chez les jeunes. On voit, en particulier, que la scolarisation dans le groupe des

15-19 ans semble jouer un rôle protecteur sur les comportements sexuels, tandis que le travail occasionnel était associé à des comportements sexuels à risque accrus dans ce groupe. Ces résultats confirment les conclusions d'études précédentes. La recherche par Hargreaves et coll. indique que parmi les jeunes Sud-Africains ruraux non mariés, âgés de 14 à 25 ans, la scolarisation protégeait en raison de la structure des réseaux sexuels associée à des comportements sexuels moins risqués [39], tandis que Behrman et De Neve et coll. soulignent que l'éducation primaire et secondaire avait des effets positifs sur l'exposition à l'activité sexuelle et sur la réduction du risque cumulatif d'infection par le VIH [40-41]. Les acquis scolaires parmi les 20-24 ans ont également un effet protecteur sur les comportements sexuels, ce qui valide les résultats de la littérature indiquant une baisse de la prévalence du VIH et des comportements sexuels moins risqués parmi les sujets les plus éduqués [42-43].

La famille constitue une autre institution sociale importante ayant un impact direct sur les comportements sexuels à risque des jeunes [44]. Nos résultats indiquent qu'avoir un seul parent parmi le groupe des plus jeunes et ne pas avoir de lien filial ou marital avec le chef de famille parmi le groupe des plus âgés étaient des variables significatives par rapport au comportement sexuel à risque. Une autre recherche indique aussi qu'une surveillance parentale perçue limitée et que le fait d'être orphelin (d'un ou des deux parents) sont associés à des comportements néfastes pour la santé [45-46].

Nos résultats réaffirment les recommandations des programmes existants pour la prévention de SSR/du VIH, y compris le besoin de se concentrer non seulement sur les interventions au niveau individuel, mais aussi sur les facteurs et les institutions sociaux et structurels qui

impactent les comportements individuels [47]. Notre recherche souligne également que certains groupes démographiques (jeunes femmes et résidents ruraux) semblent plus vulnérables aux pratiques sexuelles les plus risquées, ce qui doit inciter à l'instauration de programmes de prévention de SSR/VIH pour mieux cibler ces groupes vulnérables.

Alors que cette étude contribue aux preuves existantes sur les connaissances et la perception des risques et sur leur relation avec les comportements sexuels à risque, les résultats présentés doivent être examinés en tenant compte de quelques limitations à l'étude. Celles-ci incluent la mesure de variables socio-comportementales sur la base d'indicateurs composés, telles que les connaissances de SSR, les obstacles dans l'évaluation adéquate des attitudes et perceptions (p. ex. perception des risques), ainsi que le recours à des auto-déclarations pour les questions sensibles liées aux comportements sexuels à risque, qui pourrait entraîner une sous-estimation de leur prévalence. Pour réduire ce défi, nous avons utilisé des outils déjà testés et validés pour une utilisation dans le contexte où cette étude a été conduite. En outre, alors que nous reconnaissons qu'il y a de multiples facteurs sociaux et structurels qui influencent les comportements sexuels à risque individuel (normes sociales, accessibilité et qualité des services, pressions économiques), notre étude n'a pas exploré en profondeur ces motifs. Pour finir, la nature transversale de cette recherche inhibe notre capacité à établir la cause et l'effet de certains indicateurs d'intérêt.

CONCLUSIONS

Notre étude indique que, malgré les niveaux élevés de connaissances sur le PF, les IST et le VIH, les jeunes présentaient également un risque perçu pour soi élevé de SSR/VIH et montraient des

niveaux significatifs de comportements sexuels à risque. Ces tendances étaient significativement plus élevées parmi le groupe des 20-24 ans que parmi les 15-19 ans. Ceci semble indiquer un manque de compétences fonctionnelles parmi les jeunes pour éviter les risques liés à la SSR/le VIH.

Nous avons également découvert qu'être une femme, être déscolarisé ou travailler occasionnellement, être orphelin d'un parent, avoir des connaissances sur le PF et se percevoir comme à risque d'infection par les IST/le VIH étaient des indicateurs significatifs de comportements sexuels à risque chez les jeunes de 15-19 ans. Les facteurs associés spécifiques au groupe des 20-24 ans étaient de résider en zone rurale, de ne pas avoir d'éducation secondaire, ne pas avoir de lien filial ou marital avec le chef de famille et de se percevoir comme à risque de grossesse non désirée. Nos résultats contribuent aux preuves disponibles que les comportements sexuels à risque ne sont pas seulement fonction des connaissances sur la prévention adéquate des maladies, mais dépendent également de la capacité des jeunes à s'engager dans une attitude préventive [48], de leurs engagements positifs dans les structures sociales environnantes (école et famille) et de leur capacité à négocier des normes sociales existantes.

Étant donné qu'un Ougandais sur cinq a des comportements sexuels à risque, il y a un besoin clair d'amplifier les programmes de prévention de SSR/du VIH qui adoptent une approche holistique pour réduire les comportements sexuels à risque. Cela doit être fait en combinant des interventions au niveau individuel par des informations, une conscience des risques, un développement des compétences et une discussion sur les calculs de coûts/bénéfices impliqués

dans l'adoption de comportements préventifs liés à la SSR/au VIH avec des interventions ciblant les structures sociales impactant les comportements individuels.

LISTE DES ABRÉVIATIONS

ZD	zone de dénombrement
PF	planification familiale
VIH	virus de l'immunodéficience humaine
ODD	objectif de développement durable
SSR	santé sexuelle et reproductive
IST	infection sexuellement transmissible
UBOS	Bureau des statistiques de l'Ouganda

CONCERNANT CE SUPPLÉMENT

Cet article a été publié comme partie de l'ouvrage *Reproductive Health*, Volume 16 Supplement 1, 2019: Effective Integration of Sexual Reproductive Health and HIV Prevention, Treatment, and Care Services across sub-Saharan Africa: Where is the evidence for program implementation?
Le supplément a été publié dans le cadre d'une collaboration entre *Reproductive Health* et *BMC Public Health.* L'intégralité du contenu, avec les versions en français, en portugais et en anglais, est disponible en ligne :
https://bmcpublichealth.biomedcentral.com/articles/supplements/volume-19-supplement-1
et
https://reproductive-health-journal.biomedcentral.com/articles/supplements/volume-16-supplement-1

DÉCLARATIONS

Approbation éthique et accord de participation

L'approbation éthique de l'étude a été obtenue auprès du Comité d'éthique Mildmay des recherches de l'Ouganda (#REC REF 0312-2015) et l'approbation de la recherche auprès du

Conseil national de la science et de la technologie de l'Ouganda (SS 4018) en 2016. La participation à la recherche a été basée sur le consentement éclairé des répondants, avec l'accord oral des parents/tuteurs pour les mineurs.

Accord de publication

Non applicable.

Disponibilité des données et matériels

Les données utilisées et/ou analysées durant l'étude actuelle sont disponibles auprès de l'auteur-ressource sur demande raisonnable et avec l'autorisation du comité d'examen institutionnel (Institutional Review Board).

Conflits d'intérêts

Les auteurs déclarent ne pas avoir de conflits d'intérêts.

Financement

Cette étude a été financée par le Département du développement international (Royaume-Uni) et la Packard Foundation. Les donateurs n'ont pas participé à la conception de l'étude et/ou à la collecte, à l'analyse et à l'interprétation des données, et/ou à la rédaction du manuscrit.

Le supplément de la revue est rendu possible grâce au soutien généreux du peuple américain via la United States Agency for International Development (USAID) en partenariat avec le Fonds des Nations unies pour la population (FNUAP) et le Programme commun des Nations Unies sur le VIH/SIDA (ONUSIDA).

Les opinions exprimées dans la présente publication sont celles des auteurs et ne reflètent pas nécessairement les politiques officielles de l'USAID, du FNUAP ou de l'ONUSIDA, la mention des dénominations de ministères ou d'organismes n'implique pas non plus l'aval du gouvernement américain, du FNUAP ou de l'ONUSIDA.

Contributions des auteurs

D.T.K. a participé à la conception de l'étude, à l'analyse et à l'interprétation des données, ainsi qu'à l'ébauche de l'article. R.P.G. et R.W.M. ont participé à la conception de l'étude, à l'analyse et à l'interprétation des données, ainsi qu'à l'ébauche de l'article. Tous les auteurs ont lu et approuvé le manuscrit final.

Remerciements

Les auteurs souhaitent remercier AH Consulting pour leur contribution à la conception de l'étude et à l'acquisition des données.

RÉFÉRENCES

1. WHO, UNFPA, IPPF, UNAIDS. Sexual and reproductive health and HIV/AIDS: A framework for priority linkages. London, New York and Geneva: World Health Organization/United Nations Population Fund/International Planned Parenthood Federation/Joint United Nations Programme on HIV/AIDS; 2005.
2. WHO, UNFPA, IPPF, UNAIDS, USCF. Sexual and reproductive health and HIV linkages: Evidence review and recommendations. Geneva, New York, London, San Francisco: World Health Organization, United Nations Population Fund, International Planned Parenthood Federation, Joint United Nations Programme on HIV/AIDS, University of California San Francisco; 2009.
3. Germain A, Dixon-Mueller R, Sen G. Back to basics: HIV/AIDS belongs with sexual and reproductive health. Bulletin of the World Health Organization. 2009; doi:10.2471/BLT.09.065425.
4. Kennedy CE, Spaulding AB, Brickley DB, Almers L, Mirjahangir J, Packel L, Kennedy GE, Mbizvo M, Collins L, Osborne K. Linking sexual and reproductive health and HIV interventions: A systematic review. J Int AIDS Soc. 2010; doi:10.1186/1758-2652-13-26.

5. Druce N, Dickinson C, Attawell K, White AC, Standing H. Strengthening linkages for sexual and reproductive health, HIV and AIDS: Progress, barriers and opportunities for scaling up. London: DFID Health Resource Centre; 2006.
6. Dickinson C, Attawell K, Druce N. Progress on scaling up integrated services for sexual and reproductive health and HIV. Bulletin of the World Health Organization. 2009; doi:10.2471/BLT.08.059279.
7. IPPF, UNFPA, WHO, UNAIDS, GNP+, ICW, Young Positives. Rapid assessment tool for sexual & reproductive health and HIV linkages: A generic guide. London: IPPF, UNFPA, WHO, UNAIDS, GNP+, ICW and Young Positives; 2009.
8. UNAIDS. 90-90-90. An ambitious treatment target to help end the AIDS epidemic. Geneva: Joint United Nations Programme on HIV/AIDS; 2014.
9. UNAIDS. The Sustainable Development Goals and the HIV response. Stories of putting people at the centre. Geneva: Joint United Nations Programme on HIV/AIDS; 2017.
10. Askew I, Berer M. The contribution of sexual and reproductive health services to the fight against HIV/AIDS: a review. Reprod Health Matters. 2003;11(22):51-73.
11. Government of Uganda, UNFPA. National dialogue on SRHR/HIV and SGBV integration report, 1st - 2nd November 2017. Entebbe: Unpublished; 2017.
12. Republic of Uganda. National strategic plan for HIV&AIDS 2011/12 -2014/15. Revised. Kampala; 2012.
13. Republic of Uganda National strategy for integration of sexual and reproductive health and rights and HIV/AIDS. Kampala: Ministry of Health; 2012.
14. Government of Uganda. Presidential fast-track initiative on ending HIV & AIDS in Uganda. A presidential handbook. Kampala; 2017.
15. Inter-agency Working Group on SRH and HIV Linkages. SRH and HIV linkages compendium. Indicators & related assessment tools. London: IPPF, UNFPA, WHO; 2014.
16. Ministry of Health. Press Statement. Release of Preliminary Results of the 2016 Uganda Population HIV Impact Assessment. 2017. www.health.go.ug/download/file/fid/1505. Accessed 1 Mar 2018.
17. UBOS. Young people: The untapped resource for development. Thematic series based on the National Population and Housing Census 2014. Kampala: Uganda Bureau of Statistics; 2017.
18. IPPF, UNFPA, WHO, UNAIDS. Uganda. Rapid assessment of SRH and HIV linkages. Kampala; 2012.
19. UNDESA. Designing household survey samples: Practical guidelines. Series F No.98. New York: United Nations Department of Economic and Social Affairs; 2005.
20. Ministry of Education and Sports. National sexuality education framework. Kampala; 2018.
21. UBOS. The national population and housing census 2014 – Main report. Kampala: Uganda Bureau of Statistics; 2016.

22. Bazargan M, Kelly EM, Stein JA, Husaini BA, Bazargan SH. Correlates of HIV risk-taking behaviors among African-American college students: The effect of HIV knowledge, motivation, and behavioral skills. J Natl Med Assoc. 2000;92:391-404.
23. Helweg-Larsen M, Collins BE. A social psychological perspective on the role of knowledge about AIDS in AIDS prevention. Current Directions in Psychological Science. 1997; doi:10.1111/1467-8721.ep11512614.
24. Fisher JD, Fisher WA. Theoretical Approaches to Individual-Level Change in HIV Risk Behavior. In: Peterson JL, DiClemente RJ, editors. Handbook of HIV prevention. Boston: Springer; 2000. p. 3-55.
25. Swenson RR, Rizzo CJ, Brown LK, et al. HIV knowledge and its contribution to sexual health behaviors of low-income African American adolescents. J Natl Med Assoc. 2010; doi:10.1016/S0027-9684(15)30772-0.
26. Hulton LA, Cullen R, Khalokho SW. Perceptions of the risks of sexual activity and their consequences among Ugandan adolescents. Stud Fam Plann. 2000; doi:10.1111/j.1728-4465.2000.00035.x.
27. Gallant M, Maticka-Tyndale E. School-based HIV prevention programmes for African youth. Soc Sci Med. 2004; doi:10.1016/S0277-9536(03)00331-9.
28. Frost JJ, Lindberg LD, Finer LB. Young adults' contraceptive knowledge, norms and attitudes: Associations with risk of unintended pregnancy. Perspect Sex Reprod Health. 2012; doi: 10.1363/4410712.
29. Haider TL, Sharma M. Barriers to family planning and contraception uptake in Sub-Saharan Africa: A systematic review. Int Q Community Health Educ. 2012-2013; doi:10.2190/IQ.33.4.g.
30. Chandra-Mouli V, McCarraher DR, Phillips SJ, Williamson NE, Hainsworth G. Contraception for adolescents in low and middle income countries: needs, barriers, and access. Reprod Health. 2014; doi:10.1186/1742-4755-11-1.
31. Nalwadda G, Mirembe F, Byamugisha J, Faxelid E. Persistent high fertility in Uganda: young people recount obstacles and enabling factors to use of contraceptives. BMC Public Health. 2010; doi:10.1186/1471-2458-10-530.
32. Lindegren ML, Kennedy CE, Bain-Brickley D, Azman H, Creanga AA, Butler LM, Spaulding AB, Horvath T, Kennedy GE. Integration of HIV/AIDS services with maternal, neonatal and child health, nutrition, and family planning services. Cochrane Database Syst Rev. 2012; doi:10.1002/14651858.CD010119.
33. Millstein SG, Halpern-Felsher BL. Perceptions of risk and vulnerability. In: Fischhoff B, Nightingale EO, Iannotta JG, editors. Adolescent risk and vulnerability: Concepts and measurement. Washington D.C.: National Academies Press; 2001.
34. Fisher AW, Fisher JD, Harman J. The information–motivation–behavioral skills model: A general social psychological approach to understanding and promoting health behavior. In: Suls J, Wallston KA, editors. Social psychological foundations of health and illness. Malden: Blackwell Publishing Ltd; 2003.
35. Chang SJ, Choi S, Kim SA, Song M. Intervention strategies based on information-motivation-behavioral skills model for health behavior change: A systematic review. Asian Nursing Research. 2014; doi:10.1016/j.anr.2014.08.002.

36. Marston C, King E. Factors that shape young people's sexual behaviour: a systematic review. The Lancet. 2006; doi:10.1016/S0140-6736(06)69662-1.
37. Latkin CA, Knowlton AR. Micro-social structural approaches to HIV prevention: a social ecological perspective. AIDS Care. 2005; doi:10.1080/09540120500121185.
38. Auerbach JD, Parkhurst JO, Cáceres CF. Addressing social drivers of HIV/AIDS for the long-term response: Conceptual and methodological considerations, Glob Public Health. 2011; doi:10.1080/17441692.2011.594451.
39. Hargreaves JR, Morison LA, Kim JC, Bonell CP, Porter JD, Watts C, Busza J, Phetla G, Pronyk PM. The association between school attendance, HIV infection and sexual behaviour among young people in rural South Africa. J Epidemiol Community Health. 2008; doi:10.1136/jech.2006.053827.
40. Behrman JA. The effect of increased primary schooling on adult women's HIV status in Malawi and Uganda: Universal Primary Education as a natural experiment. Soc Sci Med. 2015; doi:10.1016/j.socscimed.2014.06.034
41. De Neve JW, Fink G, Subramanian SV, Moyo S, Bor J. Length of secondary schooling and risk of HIV infection in Botswana: evidence from a natural experiment. Lancet Glob Health. 2015; doi:10.1016/S2214-109X(15)00087-X.
42. Hargreaves JR, Bonell CP, Boler T, Boccia D, Birdthistle I, Fletcher A, Pronyk PM, Glynn JR. Systematic review exploring time trends in the association between educational attainment and risk of HIV infection in sub-Saharan Africa. AIDS. 2008; doi:10.1097/QAD.0b013e3282f2aac3.
43. Glynn JR, Caraël M, Buvé A, Anagonou S, Zekeng L, Kahindo M, Musonda R. for the Study Group on Heterogeneity of HIV Epidemics in African Cities. Does increased general schooling protect against HIV infection? A study in four African cities. Trop Med Int Health. 2004; doi:10.1046/j.1365-3156.2003.01168.x.
44. Perrino T, Gonzalez-Soldevilla A, Pantin H, Szapocznik J. The role of families in adolescent HIV prevention: A review. Clin Child Fam Psychol Rev. 2000;3(2):81–96.
45. DiClemente RJ, Wingood GM, Crosby R, Sionean C, Cobb BK, Harrington K, Davies S, Hook EW 3rd, Oh MK. Parental monitoring: association with adolescents' risk behaviors. Pediatrics. 2001 Jun;107(6):1363-8.
46. Birdthistle IJ, Floyd S, Machingura A, Mudziwapasi N, Gregson S, Glynn JR. From affected to infected? Orphanhood and HIV risk among female adolescents in urban Zimbabwe. AIDS. 2008; doi: 10.1097/QAD.0b013e3282f4cac7.
47. Dean HD, Fenton KA. Addressing social determinants of health in the prevention and control of HIV/AIDS, viral hepatitis, sexually transmitted infections, and tuberculosis. Public Health Rep. 2010; doi:10.1177/00333549101250S401.
48. Fisher JD, Fisher WA, Williams SS, Malloy TF. Empirical tests of an information-motivation-behavioral skills model of AIDS preventive behavior. Health Psychol. 1994; 13:238-250.

Tableau 1 : Répartition des participants selon les caractéristiques de contexte

Variable	15-19 ans, n=1 647	20-24 ans, n=1 078	15-24 ans, n=2 725
	Fréq. (%)	Fréq. (%)	Fréq. (%)
Résidence			
Urbaine	261 (15,8)	236 (21,9)	497 (18,2)
Rurale	1 386 (84,2)	842 (78,1)	2 228 (81,8)
Statut marital			
Jamais mariée	1 484 (90,2)	576 (53,5)	2 060 (75,7)
Déjà mariée	162 (9,8)	501 (46,5)	663 (24,3)
Sexe			
Masculin	741 (45,0)	520 (48,2)	1 261 (46,3)
Féminin	906 (55,0)	558 (51,8)	1 464 (53,7)
Statut scolaire			
Scolarisé	1 184 (72,0)	258 (24,1)	1 442 (53,1)
Déscolarisé	461 (28,0)	814 (75,9)	1 275 (46,9)
Niveau d'éducation atteint			
Aucun	18 (1,1)	21 (2,0)	39 (1,4)
Primaire partiel	833 (50,6)	422 (39,2)	1 255 (46,1)
Secondaire et plus	794 (48,3)	633 (58,8)	1 427 (52,4)
Relation au chef de famille			
Chef/conjointe	179 (10,9)	458 (42,5)	637 (23,4)
Fils/fille	1 165 (70,7)	441 (40,9)	1 606 (59,0)
Autre parent	303 (18,4)	178 (16,5)	481 (17,7)
Statut des parents			
Deux parents en vie	1 181 (71,7)	650 (60,4)	1 831 (67,2)
Un parent en vie	330 (20,0)	291 (27,0)	621 (22,8)
Deux parents morts	136 (8,3)	135 (12,5)	271 (10,0)
Activité			
Étudiant	1 003 (61,0)	203 (18,9)	1206 (44,4)
Agriculteur	263 (16,0)	369 (34,4)	632 (23,3)
Travail occasionnel	286 (17,4)	411 (38,3)	697 (25,7)
Sans emploi	91 (5,5)	91 (8,5)	182 (6,7)
Région			
Centre 1	198 (12,0)	128 (11,9)	326 (12,0)
Centre 2	164 (10,0)	103 (9,6)	267 (9,8)
Centre-Est	228 (13,8)	107 (9,9)	335 (12,3)
Est	218 (13,2)	156 (14,5)	374 (13,7)
Kampala	73 (4,4)	79 (7,3)	152 (5,6)
Karamoja	43 (2,6)	39 (3,6)	82 (3,0)
Nord	153 (9,3)	88 (8,2)	241 (8,8)
Sud-Ouest	255 (15,5)	185 (17,2)	440 (16,1)
Nil occidental	135 (8,2)	54 (5,0)	189 (6,9)
Ouest	180 (10,9)	139 (12,9)	319 (11,7)

Tableau 2 : Connaissances sur la SSR/le VIH et risque perçu par groupe d'âge

**Variable**	**15-19 ans, n=1 647**	**20-24 ans, n=1 078**	**15-24 ans, n=2 725**
	**Fréq. (%)**	**Fréq. (%)**	**Fréq. (%)**
Connaissances du PF			
Oui	1 499 (91,3)	1 056 (98,0)	2 555 (94,0)
Non	142 (8,7)	22 (2,0)	164 (6,0)
Connaissances des IST			
Oui	1 326 (82,0)	964 (91,2)	2 290 (85,6)
Non	291 (18,0)	93 (8,8)	384 (14,4)
Connaissances du VIH/SIDA			
Oui	1 379 (83,7)	970 (90,0)	2 349 (86,2)
Non	268 (16,3)	108 (10,0)	376 (13,8)
Risque perçu d'IST			
À risque	761 (46,2)	663 (61,5)	1 424 (52,3)
Pas à risque	886 (53,8)	415 (38,5)	1301 (47,7)
Risque perçu de grossesse			
À risque	657 (39,9)	567 (52,6)	1 224 (44,9)
Pas à risque	990 (60,1)	511 (47,4)	1 501 (55,1)
Comportement sexuel à risque			
Oui	245 (14,9)	373 (34,6)	618 (22,7)
Non	1 402 (85,1)	705 (65,4)	2 107 (77,3)

Tableau 3 : Facteurs associés à des comportements sexuels à risque parmi les participants âgés de 15 à 19 ans.

Variable	Analyse à deux variables				Analyse à variables multiples			
	RC	valeur p	IC 95 %		Rcc	valeur p	IC 95 %	
Résidence (réf. : rurale)	0,80	0,27	0,54	1,19	0,80	0,41	0,48	1,35
Statut marital (réf. : déjà mariée)	0,38	<0,001	0,26	0,55	0,98	0,95	0,57	1,71
Âge (continu)	1,60	<0,001	1,44	1,78	1,44	<0,001	1,26	1,64
Sexe (réf. : féminin)	1,68	<0,001	1,27	2,20	1,89	<0,001	1,37	2,61
Statut scolaire (réf. : scolarisé)	2,59	<0,001	1,96	3,42	1,44	0,16	0,87	2,41
Niveau d'éducation (réf. : secondaire et +)								
Aucun	2,16	0,15	0,76	6,17	1,59	0,48	0,44	5,70
Primaire partiel	0,95	0,69	0,72	1,24	0,89	0,55	0,62	1,29
Relation au chef de famille (réf. : enfant)								
Chef/conjointe	2,00	<0,001	1,35	2,94	1,13	0,64	0,67	1,92
autre parent	1,42	0,04	1,01	2,00	1,14	0,55	0,74	1,75
Statut des parents (réf. : tous deux en vie)								
Deux parents morts	1,54	0,07	0,96	2,45	1,20	0,52	0,69	2,12
Un parent en vie	1,80	<0,001	1,32	2,47	1,56	0,02	1,08	2,24
Activité (réf. : étudiant)								
Agriculteur	2,42	<0,001	1,69	3,46	1,11	0,70	0,65	1,91
Travail occasionnel	2,99	<0,001	2,14	4,19	2,08	<0,001	1,27	3,39
Sans emploi	1,20	0,59	0,62	2,33	0,90	0,79	0,40	1,99
Région (réf. : Centre 1)								
Centre 2	1,74	0,08	0,94	3,19	2,04	0,04	1,04	4,00
Centre-Est	0,95	0,86	0,51	1,77	1,42	0,32	0,71	2,82
Est	1,19	0,57	0,65	2,18	1,40	0,33	0,72	2,72
Kampala	0,89	0,81	0,36	2,20	1,27	0,67	0,43	3,78
Karamoja	2,55	0,03	1,10	5,91	2,49	0,08	0,91	6,78
Nord	1,49	0,22	0,79	2,81	1,49	0,29	0,72	3,09
Sud-Ouest	1,39	0,27	0,78	2,46	1,31	0,41	0,69	2,50
Nil occidental	1,82	0,06	0,97	3,43	2,35	0,02	1,17	4,73
Ouest	2,89	<0,001	1,65	5,08	3,31	<0,001	1,77	6,18
Connaissances sur la SSR et risque perçu								
Connaissances du PF (réf. : non)	3,64	<0,001	1,68	7,88	3,46	<0,001	1,47	8,14
Connaissances des IST (réf. : non)	1,87	<0,001	1,23	2,85	1,41	0,16	0,87	2,29
Connaissances du VIH/SIDA (réf. : non)	0,96	0,83	0,67	1,38	0,93	0,73	0,61	1,42
Risque perçu d'IST (réf. : aucun)	3,25	<0,001	2,42	4,36	2,88	<0,001	1,72	4,82
Risque perçu de grossesse (réf. : aucun)	2,52	<0,001	1,91	3,32	1,01	0,96	0,62	1,66

Tableau 4 : Facteurs associés à des comportements sexuels à risque parmi les participants âgés de 20 à 24 ans.

Variable	Analyse à deux variables				Analyse à variables multiples			
	RC	valeur p	IC 95 %		Rcc	valeur p	IC 95 %	
Résidence (réf. : rurale)	1,34	0,06	0,99	1,80	1,52	0,04	1,03	2,24
Statut marital (réf. : déjà mariée)	0,74	0,02	0,57	0,95	0,87	0,44	0,60	1,25
Âge (continu)	1,17	<0,001	1,08	1,27	1,12	0,02	1,02	1,23
Sexe (réf. : féminin)	1,67	<0,001	1,30	2,15	2,10	<0,001	1,56	2,81
Statut scolaire (réf. : scolarisé)	1,89	<0,001	1,38	2,60	1,27	0,42	0,71	2,28
Niveau d'éducation (réf. : secondaire et +)								
Aucun	1,58	0,31	0,65	3,80	1,97	0,17	0,74	5,22
Primaire partiel	1,28	0,06	0,99	1,66	1,46	0,02	1,07	2,01
Relation au chef de famille (réf. : enfant)								
Chef/conjointe	1,64	<0,001	1,24	2,17	1,29	0,17	0,90	1,87
Autre parent	1,62	0,01	1,12	2,33	1,55	0,04	1,03	2,34
Statut des parents (réf. : tous deux en vie)								
Deux parents morts	1,39	0,09	0,95	2,03	1,11	0,62	0,73	1,70
Un parent en vie	1,11	0,49	0,83	1,48	0,97	0,87	0,71	1,34
Activité (réf. : étudiant)								
Agriculteur	1,73	0,01	1,17	2,56	1,03	0,93	0,52	2,04
Travail occasionnel	2,56	<0,001	1,74	3,75	1,48	0,23	0,78	2,79
Sans emploi	1,44	0,20	0,82	2,51	1,01	0,97	0,47	2,19
Région (réf. : Centre 1)								
Centre 2	1,31	0,32	0,77	2,25	1,31	0,37	0,72	2,36
Centre-Est	1,39	0,23	0,82	2,35	1,42	0,23	0,80	2,53
Est	0,87	0,60	0,53	1,44	0,95	0,84	0,56	1,62
Kampala	1,05	0,88	0,58	1,89	0,60	0,16	0,30	1,22
Karamoja	0,85	0,68	0,39	1,84	0,79	0,60	0,33	1,90
Nord	0,76	0,36	0,42	1,37	0,72	0,31	0,37	1,37
Sud-Ouest	0,81	0,39	0,50	1,31	0,72	0,23	0,42	1,22
Nil occidental	1,21	0,56	0,63	2,34	1,06	0,87	0,53	2,13
Ouest	1,14	0,61	0,69	1,88	1,13	0,65	0,66	1,96
Connaissances sur la SSR et risque perçu								
Connaissances du PF (réf. : non)	1,82	0,24	0,67	4,97	1,40	0,53	0,49	4,05
Connaissances des IST (réf. : non)	1,11	0,64	0,71	1,75	1,07	0,78	0,65	1,76
Connaissances du VIH/SIDA (réf. : non)	1,42	0,12	0,91	2,21	1,55	0,08	0,95	2,53
Risque perçu d'IST (réf. : aucun)	1,27	0,07	0,98	1,65	0,84	0,45	0,53	1,32
Risque perçu de grossesse (réf. : aucun)	1,32	0,03	1,03	1,70	1,56	0,05	1,01	2,43
