# Supplementary material for: The knowledge-risk-behaviour continuum among young Ugandans: what it tells us about SRH/HIV integration
Source: BMC Public Health. 2019 May 29;19(Suppl 1):604. doi: 10.1186/s12889-019-6809-y (PMC6538546; doi:10.1186/s12889-019-6809-y)
Supplement: Supplementary file 2 — Translation of the abstract of this article into Portuguese. (PDF 271 kb) [file 12889_2019_6809_MOESM2_ESM.pdf]

BMC Public Health 2019, 19 (Suppl 1) [translated from the original publication]

O conhecimento de comportamentos sexuais de risco entre os jovens ugandeses: O que esta situação revela sobre a integração dos serviços de SSR/VIH

Raquel Palomino González,[1] Damazo Twebaze Kadengye,[2 5*] Roy William Mayega,[3 4]

[1] United Nations Population Fund (UNFPA), Kampala, Uganda, ragonzalez@unfpa.org
[2] African Population and Health Research Center, Nairobi, Kenya, dkadengye@aphrc.org
[3] School of Public Health, Makerere University, Kampala, Uganda, rmayega@musph.ac.ug
[4] AH Consulting, Kampala, Uganda
[5] D.T.K. contribution to this paper occurred while he worked with the School of Statistics and Planning, Makerere University; Kampala, Uganda and AH Consulting Ltd., Kampala, Uganda.

*Autor para correspondência:

Kadengye D.T.
E-mail: dkadengye@gmail.com / dkadengye@aphrc.org

RESUMO

Introdução: Devido a uma prevalência do vírus da imunodeficiência humana (VIH) de 2,1% entre os jovens de 15 a 24 anos, existem oportunidades para uma maior integração dos serviços de saúde sexual e reprodutiva (SSR) e de prevenção do VIH para os jovens no Uganda. Foram analisados diversos elementos que contribuem para a variação do comportamento sexual de risco entre os jovens ugandeses.

Métodos: Foi realizado um inquérito transversal às famílias, a nível nacional, entre fevereiro e março de 2016. O questionário utilizado avaliou os conhecimentos, as práticas e os comportamentos relacionados com a SSR entre jovens de 10 a 24 anos. Foi desenvolvido um indicador composto para avaliar o comportamento sexual de risco, definido como participar em relações sexuais sob a influência de álcool ou drogas, praticar atividades sexuais sem preservativo e ter múltiplos parceiros sexuais nos seis meses anteriores ao inquérito. Foi realizada uma análise exploratória para fornecer estatísticas descritivas. Também foi realizada uma regressão logística para determinar os fatores associados ao comportamento sexual de risco. Esta análise incide na subcategoria de idades entre 15 e 24 anos, composta por 2725 inquiridos.

Resultados: Os níveis de conhecimento sobre planeamento familiar (PF), infeções sexualmente transmissíveis (IST) e VIH foram elevados (acima de 82%) em todos os inquiridos. O risco percetível de IST e gravidez comunicado pelos próprios foi superior entre os inquiridos de 20 a 24 anos, com 61,5% a sentirem-se em risco de contrair uma IST em comparação com 46,2% dos inquiridos de 15 a 19 anos. 22,7% dos inquiridos indicaram que estiveram envolvidos em comportamentos sexuais de risco. Os fatores associados a comportamentos sexuais de risco entre o grupo de 15 a 19 anos incluem género, monoparentalidade, trabalho ocasional, nível de

escolaridade, conhecimento sobre PF e o risco percetível de IST/VIH pelos próprios. Para os inquiridos com idades entre 20 e 24 anos, os fatores associados significativos incluem género, nível de educação, relação com o chefe de família, local de residência e risco percetível de gravidez pelos próprios.

Conclusões: Apesar de existir um elevado nível de conhecimento geral sobre SSR/VIH, bem como um risco percetível de gravidez e do VIH, o comportamento sexual de risco entre os jovens ugandeses continua elevado. A falta de eficácia na resposta dos serviços de SSR/VIH integrados para os jovens deve ser resolvida e deve também haver uma combinação de intervenções específicas centradas na prevenção global através de informações, sensibilização para os riscos e desenvolvimento de competências a nível individual, juntamente com intervenções orientadas para estruturas sociais que afetem o comportamento individual.

Palavras-chave: SSR, VIH, Conhecimento, Perceção do risco, Comportamento sexual, Jovens, Uganda

Sobre este suplemento

Este resumo foi publicado como parte da revista científica *Reproductive Health*, Volume 16, Suplemento 1, 2019: Integração Eficaz dos Serviços de Saúde Sexual e Reprodutiva e de Prevenção, Cuidados e Tratamento do VIH na África Subsariana: Onde estão as provas da implementação do programa?
O suplemento foi publicado como uma colaboração entre as revistas científicas *Reproductive Health* e *BMC Public Health.* O conteúdo integral, incluindo as versões em francês, português e inglês, estão disponíveis online:
https://bmcpublichealth.biomedcentral.com/articles/supplements/volume-19-supplement-1 e https://reproductive-health-journal.biomedcentral.com/articles/supplements/volume-16-supplement-1
